# Pathways to care and outcomes among hospitalised HIV-seropositive persons with cryptococcal meningitis in South Africa

**DOI:** 10.1371/journal.pone.0225742

**Published:** 2019-12-12

**Authors:** Vanessa Quan, Sandra Toro-Silva, Charlotte Sriruttan, Verushka Chetty, Violet Chihota, Sophie Candfield, Anna Vassall, Alison D. Grant, Nelesh P. Govender, for GERMS-SA

**Affiliations:** 1 GERMS-SA, Division of Public Health Surveillance and Response, National Institute for Communicable Diseases, a division of the National Health Laboratory Service, Johannesburg, South Africa; 2 TB Centre, London School of Hygiene & Tropical Medicine, London, United Kingdom; 3 Centre for Healthcare-Associated Infections, Antimicrobial Resistance and Mycoses, National Institute for Communicable Diseases, a division of the National Health Laboratory Service, Johannesburg, South Africa; 4 School of Pathology, Faculty of Health Sciences, University of the Witwatersrand, Johannesburg, South Africa; 5 The Aurum Institute, Johannesburg, South Africa; 6 School of Public Health, University of the Witwatersrand, Johannesburg, South Africa; 7 The Mortimer Market Centre, Central and North West NHS Foundation Trust, London, United Kingdom; 8 Africa Health Research Institute, School of Nursing and Public Health, University of KwaZulu-Natal, Durban, South Africa; University of Minnesota, UNITED STATES

## Abstract

**Introduction:**

*Cryptococcus* causes 15% of AIDS-related deaths and in South Africa, with its high HIV burden, is the dominant cause of adult meningitis. Cryptococcal meningitis (CM) mortality is high, partly because patients enter care with advanced HIV disease and because of failure of integrated care following CM diagnosis. We evaluated pathways to hospital care, missed opportunities for HIV testing and initiation of care.

**Methods:**

We performed a cross-sectional study at five public-sector urban hospitals. We enrolled adults admitted with a first or recurrent episode of cryptococcal meningitis. Study nurses conducted interviews, supplemented by a prospective review of medical charts and laboratory records.

**Results:**

From May to October 2015, 102 participants were enrolled; median age was 40 years (interquartile range [IQR] 33.9–46.7) and 56 (55%) were male. In the six weeks prior to admission, 2/102 participants were asymptomatic, 72/100 participants sought care at a public-sector facility, 16/100 paid for private health care. The median time from seeking care to admission was 4 days (IQR, 0–27 days). Of 94 HIV-seropositive participants, only 62 (66%) knew their status and 41/62 (66%) had ever taken antiretroviral treatment. Among 13 participants with a known previous CM episode, none were taking fluconazole maintenance therapy. In-hospital management was mostly amphotericin B; in-hospital mortality was high (28/92, 30%). Sixty-four participants were discharged, 92% (59/64) on maintenance fluconazole, 4% (3/64) not on fluconazole and 3% (2/64) unknown. Twelve weeks post-discharge, 31/64 (48%) participants were lost to follow up. By 12 weeks post discharge 7/33 (21%) had died. Interviewed patients were asked if they were still on fluconazole, 11% (2/18) were not.

**Conclusions:**

Among hospitalised participants with CM, there were many missed opportunities for HIV care and linkage to ART prior to admission. Universal reflex CrAg screening may prompt earlier diagnosis of cryptococcal meningitis but there is a wider problem of timely linkage to care for HIV-seropositive people.

## Introduction

*Cryptococcus* causes 15% of AIDS-related deaths [1] and in South Africa, is the dominant cause of adult meningitis [2]. Most cases of cryptococcal meningitis (CM) occur among persons with advanced immunosuppression (i.e. CD4+ T-cell [CD4] count <200 cells/μl) [3]. CM-related mortality is high [1], ranging from 40% at two weeks among those treated with optimal antifungal regimens and antiretroviral therapy (ART) [4] to 60% in routine care [5–7]. Continuing high CM incidence in many sub-Saharan African countries indicates a failure of timely ART initiation prior to advanced HIV disease. High CM mortality is partly a consequence of entering care with severe immunosuppression and advanced cryptococcal disease, but also a failure of integrated care following CM diagnosis. In Cape Town, for instance, 73% of patients admitted with CM had already received an HIV diagnosis a median of four months earlier [8]. A study from Ghana in 2012–13 reported high in-hospital mortality (222/547, 40.6%) among HIV-seropositive adults with CM, 190 (85.6%) of whom were previously known to be HIV-seropositive. A majority (141/222, 63.5%) of those who died were ART-naive [9].

Hospital-based care of patients with CM puts substantial pressure on a health system. CM is a frequent cause of hospitalisation in South Africa and Uganda [10, 11]. Routine reflex laboratory cryptococcal antigen (CrAg) screening for HIV-positive persons with a CD4 count <100 cells/μl (i.e. automatic CrAg testing of remnant settled plasma specimens) had been implemented by the National Health Laboratory Service (NHLS) CD4 laboratories as a pilot programme in two provinces from 2012 and expanded across South Africa in 2016 [12]. Apart from improving case detection, management and outcomes associated with cryptococcosis, this programme was also expected to partially shift CM diagnosis to primary care level. The CrAg screening results were returned to the clinicians rather than to the microbiology laboratory, lumbar puncture was recommended for patients with a positive CrAg screen.

South Africa’s public healthcare system is tiered. Primary healthcare clinics are run by nurses with infrequent to regular visits from consulting doctors. All such clinics have a designated referral hospital. During the scale-up of the national ART programme, the care of a large proportion of HIV-seropositive individuals was shifted to nurses trained to initiate and continue ART at these clinics [13]. Nurses refer ill patients for hospital care. Lumbar punctures are also not performed by nurses so any patient with symptoms of meningitis would be sent to a referral hospital [14]. Conversely, patients who are not too ill and seek initial care at a hospital can be sent to their nearest clinic to reduce the load on the hospital (hospital transport is arranged). Acutely-ill patients who arrive at a hospital are seen. In our study, patients who sought care directly at the hospital and were seen would have been acutely ill with CM and admitted. Patients who sought care from a private doctor and who could not afford private hospital care would have been referred to a public hospital for management. ART and maintenance fluconazole medicines can be collected at the nearest clinic rather than the hospital at which CM was diagnosed, but clinic stock-outs do occur. At the time of this study, patients were not sent reminders to collect medication.

South Africa has implemented interventions for CM prevention, early diagnosis and improved management. Amphotericin B deoxycholate has been available for routine CM treatment since 2005, though prevention, monitoring and management of toxicities related to this antifungal agent should be improved further [15]. A provider-initiated CrAg screening pilot was established in the Western Cape Province in 2012 [16] and a reflex laboratory CrAg screening pilot was implemented in Gauteng Province, Free State Province and KwaZulu-Natal Province between 2012 and 2016. National CrAg screening was implemented in October 2016 [17]. With evidence from the ACTA trial [4] and recommendations by WHO that 5-FC be included in a CM treatment regimen, a 5FC clinical access programme for CM started in 2018; 5-FC is still not registered in SA [17]. ART is now universally recommended in SA [18] and the HIV test and treat initiative has the potential to decrease the number of patients seeking care very late with CM.

The aim of this study was to evaluate pathways prior to hospital care, missed opportunities to link to care or access ART and post-discharge outcomes among patients hospitalised with cryptococcosis during an early phase of CrAg screening in South Africa.

## Methods

We performed this cross-sectional study nested within a laboratory-based surveillance programme (GERMS-SA) for cryptococcosis. The study was conducted at five public-sector urban hospitals in South Africa’s Gauteng province from May to October 2015, before national roll-out of reflex laboratory CrAg screening. Three hospitals were selected from districts where reflex CrAg screening occured. Two hospitals were located in a district where reflex screening had not yet been implemented. At the time of our study, patients were only eligible for ART with a CD4 count ≤500 cells/μl [19].

As part of the GERMS-SA surveillance programme, surveillance nurses visited the hospital microbiology laboratory daily to check for positive cryptococcal test results. They were not involved in the reflex CrAg screening programme. An episode of cryptococcosis was defined by a positive cerebrospinal fluid (CSF) India ink test, a positive CSF or blood CrAg test or culture of *Cryptococcus neoformans* or *Cryptococcus gattii* from any specimen. We assumed an episode was recurrent if we found a record of a prior laboratory-confirmed episode or prior antifungal treatment for CM within 30 days. For this nested study, participants were eligible for enrolment if they were ≥18 years old, admitted to the study hospitals with a first or recurrent episode of laboratory-confirmed or presumed CM (e.g. symptoms of meningitis plus a positive blood CrAg test and on amphotericin B) and provided informed consent. Persons with CM who were identified after their admission through routine surveillance audits of the NHLS laboratory information system (LIS), referred to as “audit cases”, were not available for interview and were therefore excluded. For potential participants with a decreased level of consciousness, surveillance nurses visited the patient during their hospital stay to check for an improvement; if no improvement occurred, or if the potential participant died in hospital, their next of kin was approached and offered participation in the study. Written informed consent was obtained from the participant or the next of kin after an explanation by the study nurse. Interviews were conducted by surveillance nurses in the preferred language of the participant or next of kin. Information collected included demographic details, contacts made with the health system during the course of the illness, processes which resulted in the current hospital admission, barriers and facilitators to accessing health care, management of the episode of cryptococcosis in hospital and in-hospital outcomes. Interviews were supplemented by a prospective review of medical and laboratory records. Approximately 12 weeks after hospital discharge, participants or their next of kin were contacted telephonically to assess vital status.

The case report forms were piloted at a large academic hospital and relevant changes were then made. Data were captured on the standardised case report form, checked for correctness and captured in MS Excel. This dataset was merged with the GERMS-SA surveillance database using participant identifiers so that routine surveillance information (which includes laboratory data and clinical information) and additional information from this study were available for each participant. Stata version 14 (StataCorp Inc., College Station, Texas, USA) was used for a descriptive epidemiological analysis. We used the Wilcoxon rank sum test to compare medians and Fishers’ exact test to compare categorical variables.

Study approval was obtained from the research ethics committees of the University of the Witwatersrand and the London School of Hygiene & Tropical Medicine.

## Results

### Participants

**Fig 1:**
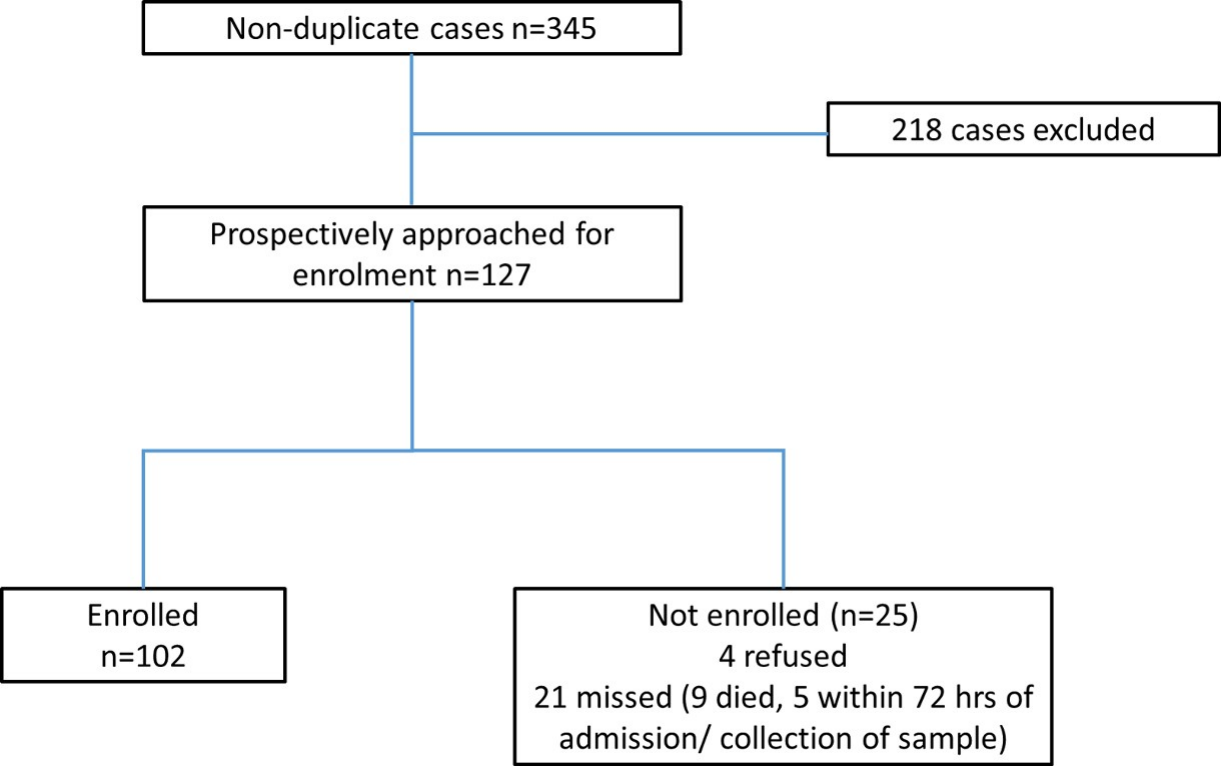
Screening and enrolment of participants.

During the study period from May through to October 2015, 345 cases of cryptococcosis were detected through surveillance; 218 cases detected through audit were excluded (participants not interviewed through routine GERMS-SA surveillance and thus very limited information was available) (Fig 1). Of 127 patients who were identified prospectively as eligible for screening, 102 participants were enrolled. There were no differences in age or sex between cases detected between those participants who were enrolled and those not enrolled (Table 1).

**Table 1:** Characteristics of cases detected through surveillance audit, enrolled participants and those who were not enrolled.

Characteristic	Enrolled (n = 102)	Not enrolled (n = 25)	p-value#	Audit cases (n = 218)
Median age in years, (IQR)	39.9 (33.9–46.7)	40.4 (31.6–44.9)	0.69	38.8 (32.8–45.2) (n = 172)
Male sex, n/N (%)	56/102 (55)	19/25 (76)	0.06	112/202 (55)
Specimen type, n/N (%)			0.14	Not available
CSF	87/102 (85)	25/25 (100)		
Serum/ plasma	8/102 (8)	0/25 (0)		
Blood culture	7/102 (7)	0/25 (0)		
Cryptococcal antigen test positive, n/N (%)	90/99 (91)*	19/25 (76)**	0.06	Not available
Cryptococcal culture positive, n/N (%)	78/85 (92)***	12/14 (86)***	0.61	Not available
CSF India ink positive, n/N (%)	76/77 (99)**	24/24 (100)***	0.80	Not available

# p-value was comparison of those enrolled vs not enrolled

*3 had no result,

**6 had no result,

*** rest had no data

All 127 participants had a diagnosis of CM (either CSF-confirmed CM or a positive blood specimen plus clinical signs of CM). Of 102 enrolled, 89 were first episodes and 13 recurrent and of 25 not enrolled, 21 were first episodes and four recurrent. None of the recurrent cases had a CSF CrAg test as the only positive test. Among the 102 enrolled participants, information was captured by participant interview in 70% (n = 71) and the remainder by next of kin interview. Eighty-five (83%) participants were from hospitals where CrAg screening was not operational and 17 from sites with routine CrAg screening. Of the 102 participants, only one had a screening blood CrAg test ≥14 days before her CM diagnosis, which may have prompted earlier CM diagnosis and treatment.

### Health-care seeking behaviour six weeks prior to admission

#### Public clinic information

Of the 102 participants, 100 had symptoms in the six weeks prior to their admission. The majority (72/100; 72%) first sought healthcare for symptoms at a public-sector clinic or hospital where a minimal or no fee is levied. A large number of visits were made to various health care providers before participants were admitted but mostly at public clinics (median 1; range 1–7 visits) (Table 2).

**Table 2:** Participants’ health-seeking behaviour and visits to health-care facilities in the six weeks preceding admission.

Healthcare facility	First place sought care for symptoms within 6 weeks of admission* (n = 100) n (%)	Attended for any care n (%)**	Total number of visits for any care in 6 weeks prior to admission	Range and median number of visits for any care in 6 weeks prior to admission
Public clinic	50 (50)	57 (57)	84	1–7; 1
Public hospital	22 (22)	30 (30)	34	1–3; 1
Private doctor	14 (14)	19 (19)	23	1–3; 1
Pharmacy	8 (8)	35 (35)	55	1–5; 1
Traditional healer	2 (2)	8 (8)	12	1–3; 1
Supermarket	4 (4)	N/A	N/A	N/A
Private hospital casualty	0	0	0	N/A

*2 patients had no symptoms and did not seek care but still had laboratory-confirmed meningitis

** more than one place where care was sought

Of 38 participants who did not first attend a primary healthcare clinic in the public sector and who answered the question, “what reasons best describe why you did not visit a public for your symptoms?”: 17/38 (45%) went directly to hospital because they were so ill, 10/38 (26%) preferred to look for help elsewhere, 5/38 (13%) were unsure that they needed to get help and 6/38 (16%) provided no reason or other reasons (e.g. did not know where the nearest clinic was, was concerned about the long clinic wait, was afraid that they would be admitted to hospital). Of 57 who visited a public clinic, 43 (75%) visited only one clinic and 48 (84%) visited the clinic closest to them; the nine who attended a clinic further from home had the following reasons: they were treated better (n = 2), they wanted privacy (n = 2), the participant was turned away from closest clinic (n = 1), there was a shortage of treatment at the nearer clinic (n = 1) and the participant was referred from work (n = 1).

#### Other health care providers

Of 100 participants, 16 paid for healthcare (at a private doctor or traditional healer) and 12 went to a pharmacy or supermarket to buy medicines (Table 2). No participants sought care at a private hospital casualty department. The median time from first seeking care to admission was four days (IQR, 0–27 days) among 99 participants with data.

### HIV testing and missed opportunities

**Fig 2:**
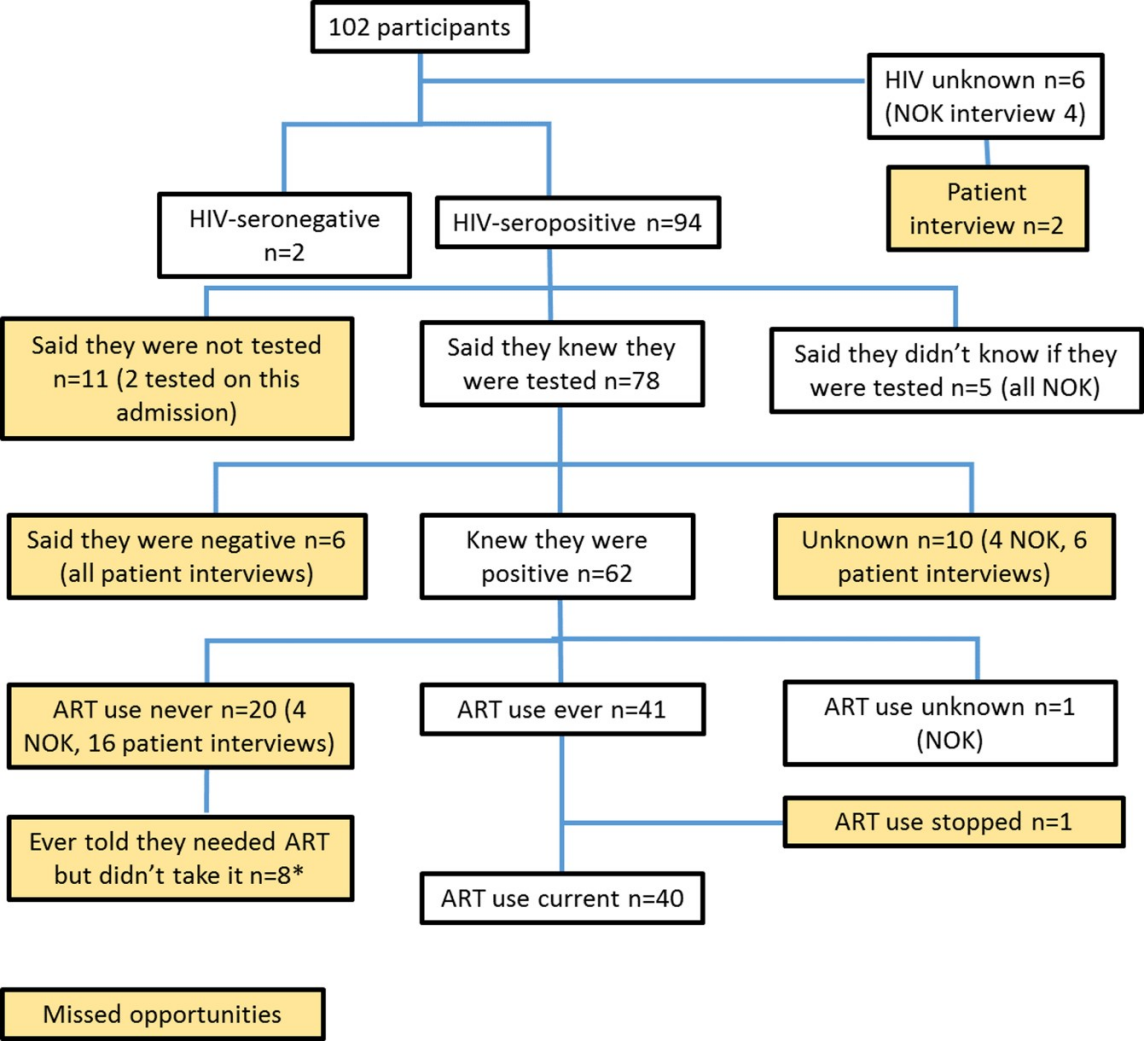
HIV testing, antiretroviral treatment and missed opportunities. Legend: NOK = next of kin, *Reasons for not starting ART: afraid of side effects (n = 2), no food (n = 1), dropped out/ did not complete adherence counselling (n = 3), relocated (n = 1), on some treatment (n = 1).

Of 102 participants, 94 were HIV-seropositive, two were HIV-seronegative and six had an unknown HIV status (four were next of kin interviews, the other two patients did not have an HIV result). On interview, 78/94 (83%) knew that they or their relative had been tested for HIV, 11 said they had never been tested (two were tested on this admission, one of whom had been previously treated for pulmonary TB) and for five, this information was unknown (all next of kin interviews). Of the 78 who knew they were tested, 62 (79%) said that they or their relative was HIV-seropositive, six said they were HIV-seronegative and 10 did not know (Fig 2 –missed opportunities for HIV testing at any health-care contact are highlighted in yellow boxes).

The date of “first HIV test” recorded was available for 67 patients. The time from when they were first tested for HIV to this admission for CM ranged from 0 days to 20 years (median 271 days, IQR 76 days to 40 months). There were 41 participants who tested at a public clinic, 36 knew whether a CD4 count had been done: seven said they had no CD4 count done and 29 (80%) said they had a CD4 count performed but only 17 (58%) returned for their result. Reasons provided for not returning for their CD4 count results (n = 10) included: three felt too ill to come back, two relocated, two did not trust the result, two did not know to return, one could not wait for the result.

At the time of the hospital diagnosis of cryptococcosis, there were 59 participants with a CD4 count ranging from 1–234 cells/μl (median 34, IQR 18–75); only three had a CD4 count >200 cells/μl; five of those participants knew their CD4 count six months prior to their diagnosis of cryptococcosis.

### ART and linkage to care

Fig 2 also shows that of 62 participants who knew they were HIV-seropositive, 41 reported ART use (one had stopped because the medicines made him/her feel ill but the other 40 were currently on ART). Thirty-six knew the approximate date they had started ART and this ranged from seven days to 110 months (median 218 days). Twenty participants had never been on ART: eight had been told they required it but did not start (three did not complete adherence counselling, two were afraid of side effects and one each relocated, reported having no food, and being on other treatment); four reported they had not been told they needed ART and one was not eligible because of a high CD4 count. Thirty-two participants had recorded dates for “first HIV test” and ART initiation. The interval between HIV testing and starting ART ranged from 0–4202 days (11.5 years) (median 31 days, IQR 0–266 days).

### Post-discharge follow-up of prior episodes of cryptococcosis and other missed opportunities

Thirteen of eighty-seven reported a previous episode of CM (number of episodes ranged from one to five [median 1, IQR 1–2]) and 11 of these knew they had received treatment for meningitis (corroborated with documentation from previous medical records). Nine of 11 participants reported receiving fluconazole on prior discharge (two never received) for a period ranging from one week to 30 weeks (median 4 weeks, IQR 2–6 weeks). All nine stopped their fluconazole for one of the following reasons: a healthcare worker stopped treatment for three, three did not know to continue after their initial course, three stopped on their own (two felt better and one felt ill on fluconazole). Of those 13 participants with a recurrent CM episode, information on ART was available for eight of them and five were on ART.

### Management of cryptococcosis in hospital

We collected treatment information during the admission for CM for 81 patients, 75 (93%) of whom received both amphotericin B (AmB) and fluconazole, five (6%) received AmB only and one received fluconazole only (800 mg twice daily for 10 days). Of the 77 who were on fluconazole (with a known duration of treatment), treatment ranged from 1–45 days (median 16 days, IQR 10–18). Of 78 patients who received AmB with a known duration of treatment, this ranged from 1–27 days (median 14 days, IQR 9–14 days).

### Outcomes and management post-hospitalisation

From the information available, we determined in-hospital outcome for 92 participants: 64 (70%) recovered and 28 (30%) died in hospital. For 26 of 28 participants who died in hospital (with date of death recorded), median time to death was 9.5 days (IQR 17.5 days, range 0–64 days). The only participant with CrAg screening done before diagnosis was a 40-year-old woman who had a blood CrAg test on 26 June 2015, followed by transfer to an academic hospital on 4 July where she started fluconazole that day and AmB the following day. A lumbar puncture (with an opening pressure of 40 cm CSF) was performed on 7 July confirming CM. She received treatment until 14 July and was discharged on fluconazole; she died a month later.

**Fig 3:**
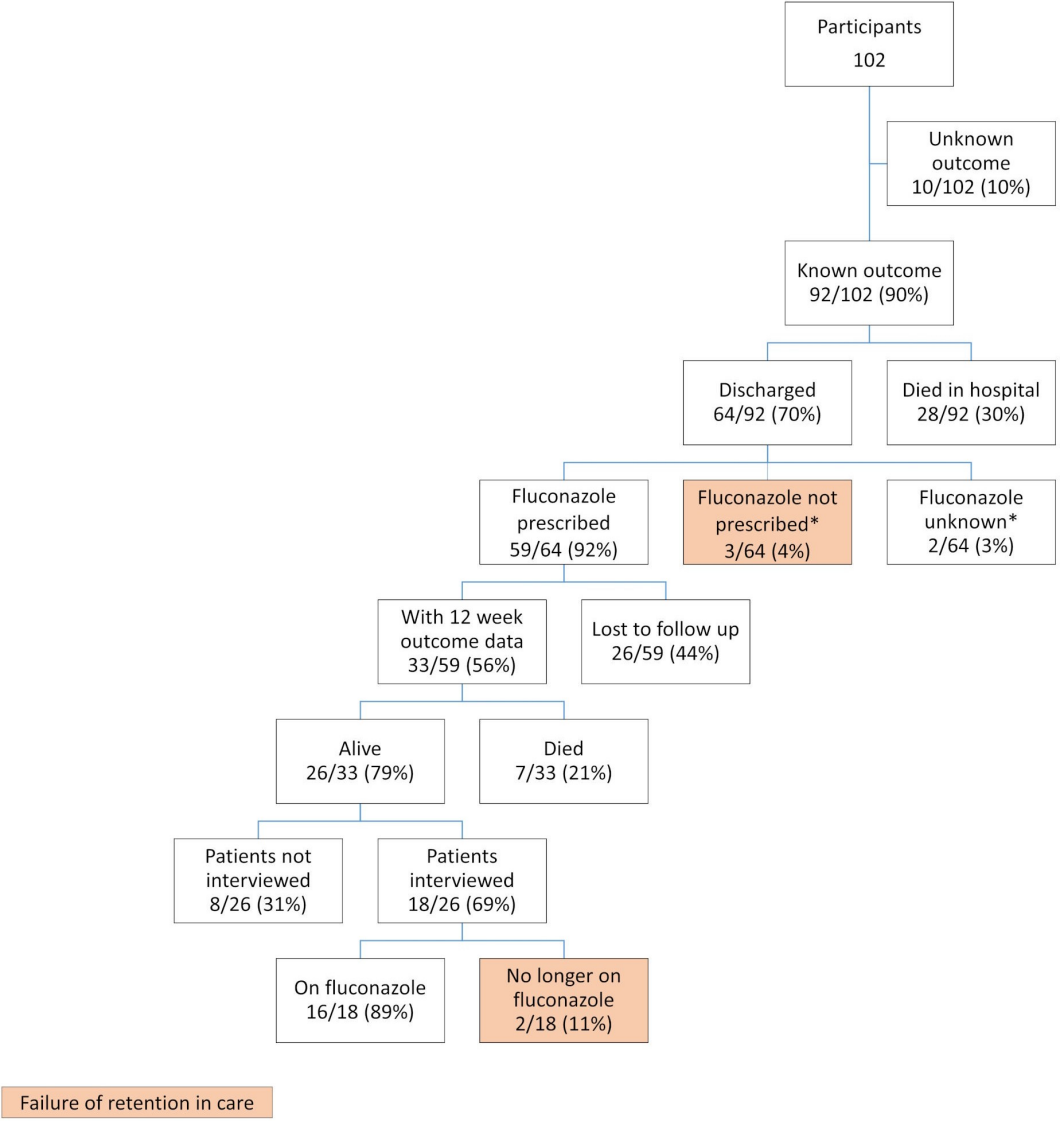
Outcomes during admission, post discharge and failure of retention in care. Key: NOK = next of kin. *lost to follow up.

Sixty-four participants were discharged alive from hospital, 59 (92%) of whom were discharged on fluconazole, three (4%) were not, two (4%) were unknown. Of the 64 participants who were discharged alive, 31 (48%) were lost to follow up at 12 weeks. Thirty-three of 59 (56%) participants discharged on fluconazole had outcome data, among whom 7/33 (21%) had died. Only participants who were interviewed were asked whether they were still taking fluconazole. Sixty-nine per cent (18/26) were interviewed: 2/18 (11%) had stopped the fluconazole; reasons were not explored. (Fig 3). Assuming that all who were lost to follow up either survived or died, the mortality ranged from 41% to 72% at 12 weeks.

## Discussion

In this study of care pathways and outcomes in CM, we have described the difficulties that very ill patients faced when navigating the complex South African healthcare system. Other SSA countries face similar challenges with a large burden of HIV/AIDS diverting scarce resources from competing health needs. Healthcare system strengthening in SSA is central to improve delivery of care for HIV/AIDS, co-morbidities and other priority conditions. Obstacles to adequate delivery of care must be pinpointed within a health care system before they can be overcome [20–22]. The literature on patient pathways for CM is very sparse. In 2011, Lessels et al described inadequate HIV status confirmation among patients seeking care for CM and a high rate of loss to follow-up once discharged, highlighting poor linkage between hospital services and PHC clinics providing ongoing HIV care and treatment in rural KwaZulu-Natal [6].

Prior to CM diagnosis, we identified numerous missed opportunities relating to HIV testing, including participants who did not know their HIV status, those who said they had never been tested and those who had been tested but thought they were HIV-seronegative. Reasons for delays in HIV testing have been explored in prior studies: social stigma, a perception of low HIV risk until they have physical symptoms, uncertainty about ART availability and side effects remain issues to be dealt with [23–26].

Linkage to HIV care is a challenging component of the HIV care cascade. There were missed opportunities related to pre-CM ART initiation, including lack of CD4 result follow up, incomplete ART adherence counselling and ART interruption. National laboratory-based cryptococcal disease surveillance data (GERMS-SA programme) in South Africa shows that more than half of patients admitted with cryptococcosis are ART-experienced (53% in 2015, 57% in 2016 and 60% in 2017) [27–29]. Earlier HIV testing (including testing by pharmacists, traditional healers and self-testing) could facilitate earlier HIV diagnosis and referral for ART. In a rural South African setting, home-based HIV-testing was well accepted although coverage for men was lower, but linkage to care took up to a year to achieve [30]. Steele and colleagues found that only 36% of those newly diagnosed after community-based HIV-testing in KwaZulu-Natal between 2013 and 2017 were linked to care within 6 months [31].

HIV self-testing (which has been included in the South African National Strategic Plan for HIV, STIs and TB, 2017–2022 [18]) is a relatively new initiative which holds promise to improve knowledge of HIV status [32–34], though advanced HIV disease will only be prevented if people with positive test results link to effective ART.

Retention in ART care has many challenges including navigation through the healthcare system, individual perceptions and medication-related issues. Not all clinics are open 24 hours, waiting times may be very long and patients are concerned about loss of confidentiality. Mental health and alcohol/ substance abuse have further been found to impact retention in ART care [35, 36]. Strengthening follow up of patients at a community level, including the use of mobile technology, may improve retention although these are costly and labour-intensive approaches [26, 37, 38].

The national South African CrAg reflex laboratory screening programme has increased detection of cryptococcosis. From October 2016 to September 2017, 276 125 patients with a CD4 count <100 cells/μl were screened nationally (95% coverage) and 15 757 (5.7%) were identified with cryptococcal antigenaemia. The effectiveness of the programme in terms of mortality reduction is being evaluated through the NICD’s CAST-NET project [17]. In our study (which was conducted during the early phase of reflex CrAg screening) only one patient had a reflex CrAg test which may have led her to be diagnosed with CM earlier. She did, however, die which illustrates the complexity of the pathway to improved outcomes. This point is reinforced by the finding that the median time between first seeking care and admission was only four days suggesting that it may be hard to improve outcomes by intervening at this point. What is really needed are further efforts to prevent advanced disease by earlier ART initiation.

In-hospital management of CM included amphotericin B for the vast majority of participants. Despite this, the in-hospital mortality was high (30%). This may be related to use of less-effective antifungal regimens in a resource-limited setting, complications associated with healthcare-associated infections, toxicity of antifungal medicines and inadequate management of raised intracranial pressure. A new WHO guideline for cryptococcal disease (March 2018) includes a one-week short course of amphotericin B deoxycholate and 5-flucytosine followed by one week of fluconazole [3]. This regimen reduces mortality by 38%, is safer and reduces the risk of anaemia (a frequent complication of antifungal treatment) by 69%, when compared to the standard two-week regimen. Most participants were appropriately discharged on consolidation/ maintenance doses of fluconazole, though there were a few missed opportunities too.

Following discharge from hospital, patients reported difficulties with continuing antifungal treatment and linking to ART at an appropriate time, particularly related to an inadequate healthcare worker explanation of the need for long-term antifungal treatment for CM. Adherence is a key challenge in long-term treatment and should be promoted by good counselling and a caring environment before beginning ART and antifungal therapy. Of our study participants who were discharged on fluconazole and available for the 12-week follow up, two had discontinued fluconazole, though reasons were not pursued. Continued counselling and education throughout treatment, pill counts done at every visit, assisting with practical reminders, helping to problem solve, understanding the challenges of long-term therapy, encouraging disclosure to a friend or family so there is support and regular support groups are all useful in adherence. Improving health systems to avoid treatment stock outs, to make care more accessible, possibly longer opening hours, reducing waiting times ease of collecting medication in the community rather than only the clinic, are also necessary [26, 39]. Systems to reduce patient loss-to-follow-up is key to managing long-term treatment as well as tracing patients who are lost to follow-up and restarting them on care.

Information post discharge was scarce; however, among patients for whom there was data, most were alive at 12 weeks. This is a biased estimate since “loss to follow-up” of HIV positive patients usually includes many unreported deaths. Assuming all who were lost to follow up died, mortality would have been 72% at 12 weeks. A study done in 2009, in a Johannesburg urban hospital, showed poor post-discharge linkage to care for TB/HIV and late presentation at a primary clinic for follow up. Twenty three per cent (137/456) of patients failed to link to TB care, 30% (138/456) who were linked arrived late (>1 week). Thirty eight per cent (186/486) of patients with HIV-associated TB failed to link to HIV care, but 32% (96/300) who were linked sought care more than 30 days late [40].

Strengths of this study include its uniqueness being nested within an ongoing, active surveillance system. We enrolled 80% of potentially eligible participants and data were collected by prospective interview. The use of next of kin interviews helped to increase representation of the sickest patients in the data set. Limitations include missing data because of next of kin interviews, patients not knowing enough about their health, incomplete medical records, and incomplete recollection of dates of when the patient was ill or when he/she was on previous treatment. The follow-up study did not reach all patients, only half who recovered were contacted.

## Conclusions

We have documented common problems in the pathway to care for patients with CM. We documented many missed opportunities for HIV testing, ART initiation and linkage to post-discharge HIV care. CrAg screening may improve CM case-finding and the availability of 5-flucytocine regimen may reduce CM mortality but the incidence of CM is only likely to decrease substantially when more people know their HIV status and start ART earlier.

## Supporting information

S1 Dataset (XLSX)
